# Supplementary material for: Functional Fruit Trees from the Atlantic and Amazon Forests: Selection of Potential Chestnut Trees Rich in Antioxidants, Nutrients, and Fatty Acids
Source: Foods. 2023 Dec 9;12(24):4422. doi: 10.3390/foods12244422 (PMC10743210; doi:10.3390/foods12244422)
Supplement: Supplementary file 1 [file foods-12-04422-s001.zip › Supplementary figures.pdf]

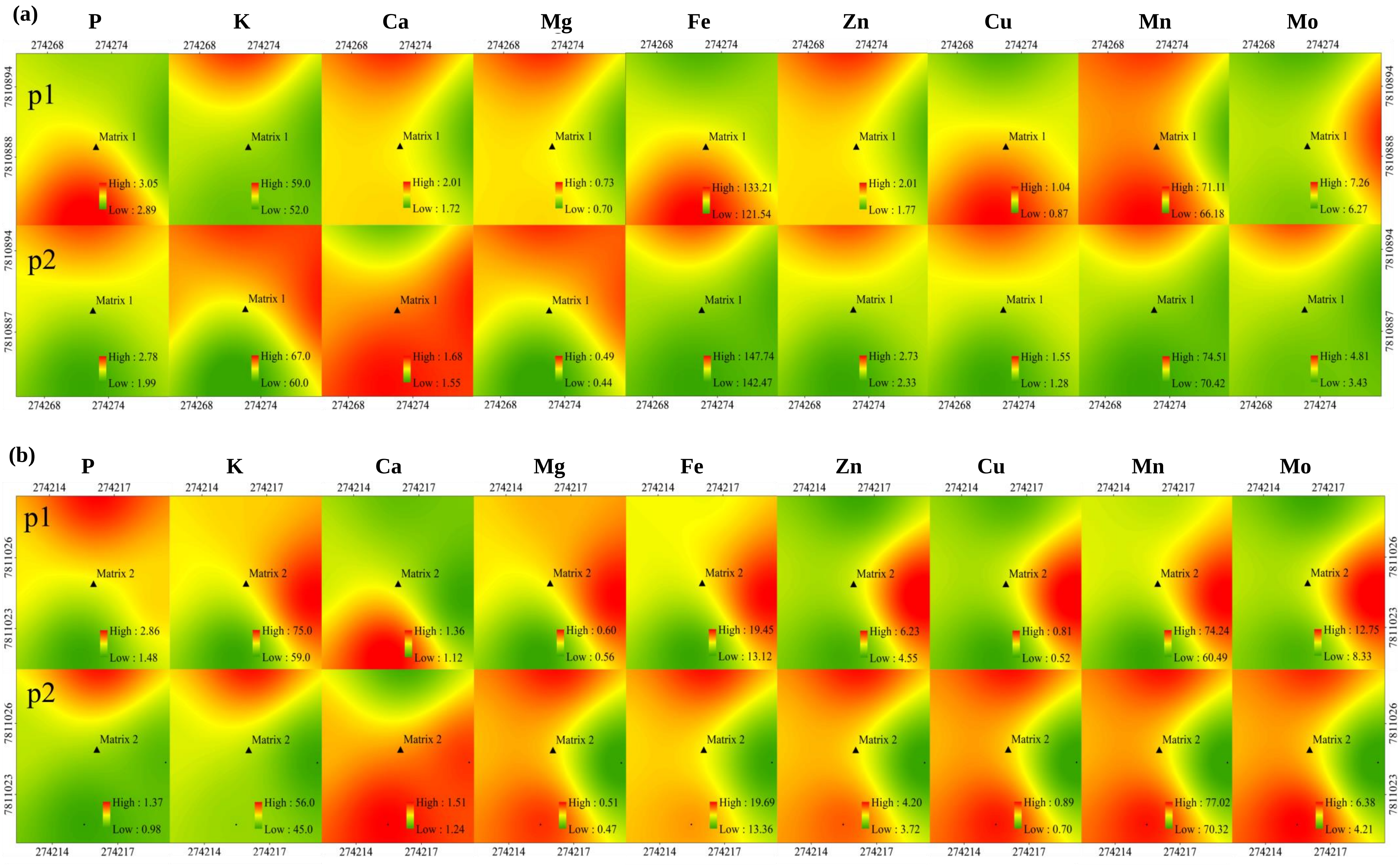
(a)
P
K
Ca
Mg
Fe
Zn
Cu
Mn
Mo
274268
274274
7810894
7810888
7810887
p1
p2
Matrix 1
High : 3.05
Low : 2.89
High : 59.0
Low : 52.0
High : 2.01
Low : 1.72
High : 0.73
Low : 0.70
High : 133.21
Low : 121.54
High : 2.01
Low : 1.77
High : 1.04
Low : 0.87
High : 71.11
Low : 66.18
High : 7.26
Low : 6.27
High : 2.78
Low : 1.99
High : 67.0
Low : 60.0
High : 1.68
Low : 1.55
High : 0.49
Low : 0.44
High : 147.74
Low : 142.47
High : 2.73
Low : 2.33
High : 1.55
Low : 1.28
High : 74.51
Low : 70.42
High : 4.81
Low : 3.43
(b)
P
K
Ca
Mg
Fe
Zn
Cu
Mn
Mo
274214
274217
7811026
7811023
p1
p2
Matrix 2
High : 2.86
Low : 1.48
High : 75.0
Low : 59.0
High : 1.36
Low : 1.12
High : 0.60
Low : 0.56
High : 19.45
Low : 13.12
High : 6.23
Low : 4.55
High : 0.81
Low : 0.52
High : 74.24
Low : 60.49
High : 12.75
Low : 8.33
High : 1.37
Low : 0.98
High : 56.0
Low : 45.0
High : 1.51
Low : 1.24
High : 0.51
Low : 0.47
High : 19.69
Low : 13.36
High : 4.20
Low : 3.72
High : 0.89
Low : 0.70
High : 77.02
Low : 70.32
High : 6.38
Low : 4.21

(c)

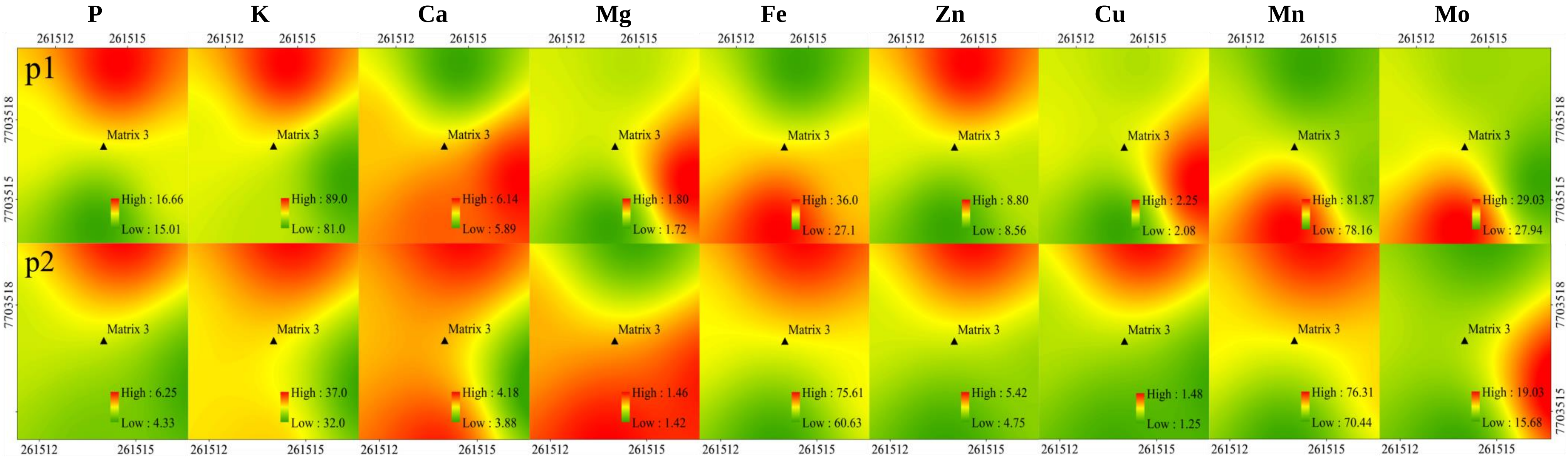
P
K
Ca
Mg
Fe
Zn
Cu
Mn
Mo
261512
261515
7703518
7703515
p1
p2
Matrix 3
High : 16.66
Low : 15.01
High : 89.0
Low : 81.0
High : 6.14
Low : 5.89
High : 1.80
Low : 1.72
High : 36.0
Low : 27.1
High : 8.80
Low : 8.56
High : 2.25
Low : 2.08
High : 81.87
Low : 78.16
High : 29.03
Low : 27.94
High : 6.25
Low : 4.33
High : 37.0
Low : 32.0
High : 4.18
Low : 3.88
High : 1.46
Low : 1.42
High : 75.61
Low : 60.63
High : 5.42
Low : 4.75
High : 1.48
Low : 1.25
High : 76.31
Low : 70.44
High : 19.03
Low : 15.68

(d)

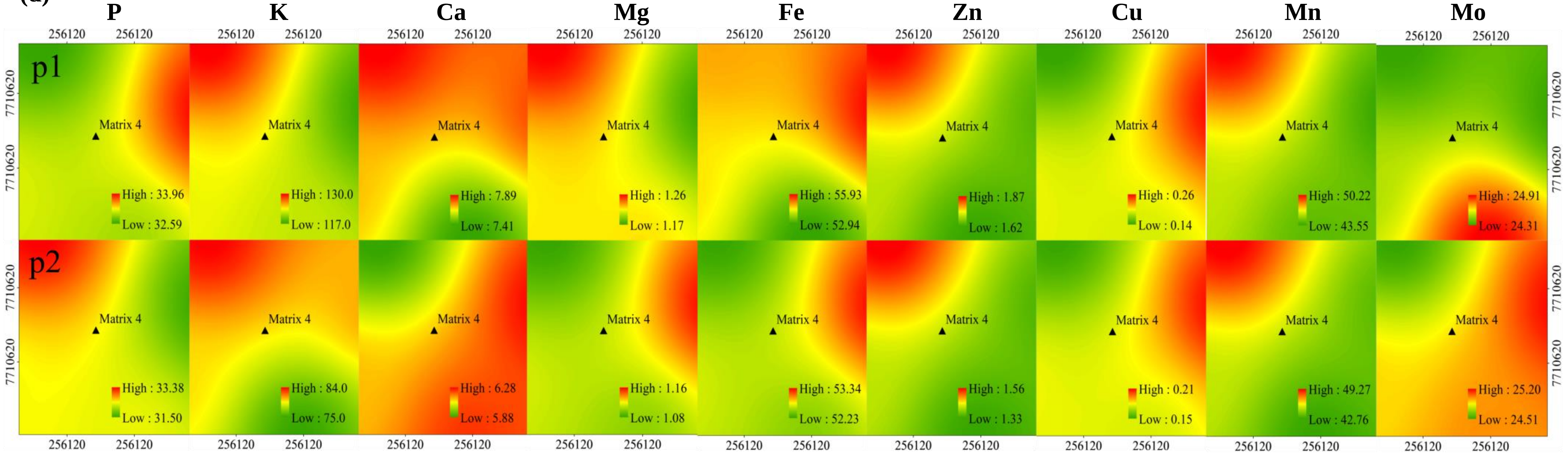
P
K
Ca
Mg
Fe
Zn
Cu
Mn
Mo
256120
7710620
p1
p2
Matrix 4
High : 33.96
Low : 32.59
High : 130.0
Low : 117.0
High : 7.89
Low : 7.41
High : 1.26
Low : 1.17
High : 55.93
Low : 52.94
High : 1.87
Low : 1.62
High : 0.26
Low : 0.14
High : 50.22
Low : 43.55
High : 24.91
Low : 24.31
High : 33.38
Low : 31.50
High : 84.0
Low : 75.0
High : 6.28
Low : 5.88
High : 1.16
Low : 1.08
High : 53.34
Low : 52.23
High : 1.56
Low : 1.33
High : 0.21
Low : 0.15
High : 49.27
Low : 42.76
High : 25.20
Low : 24.51

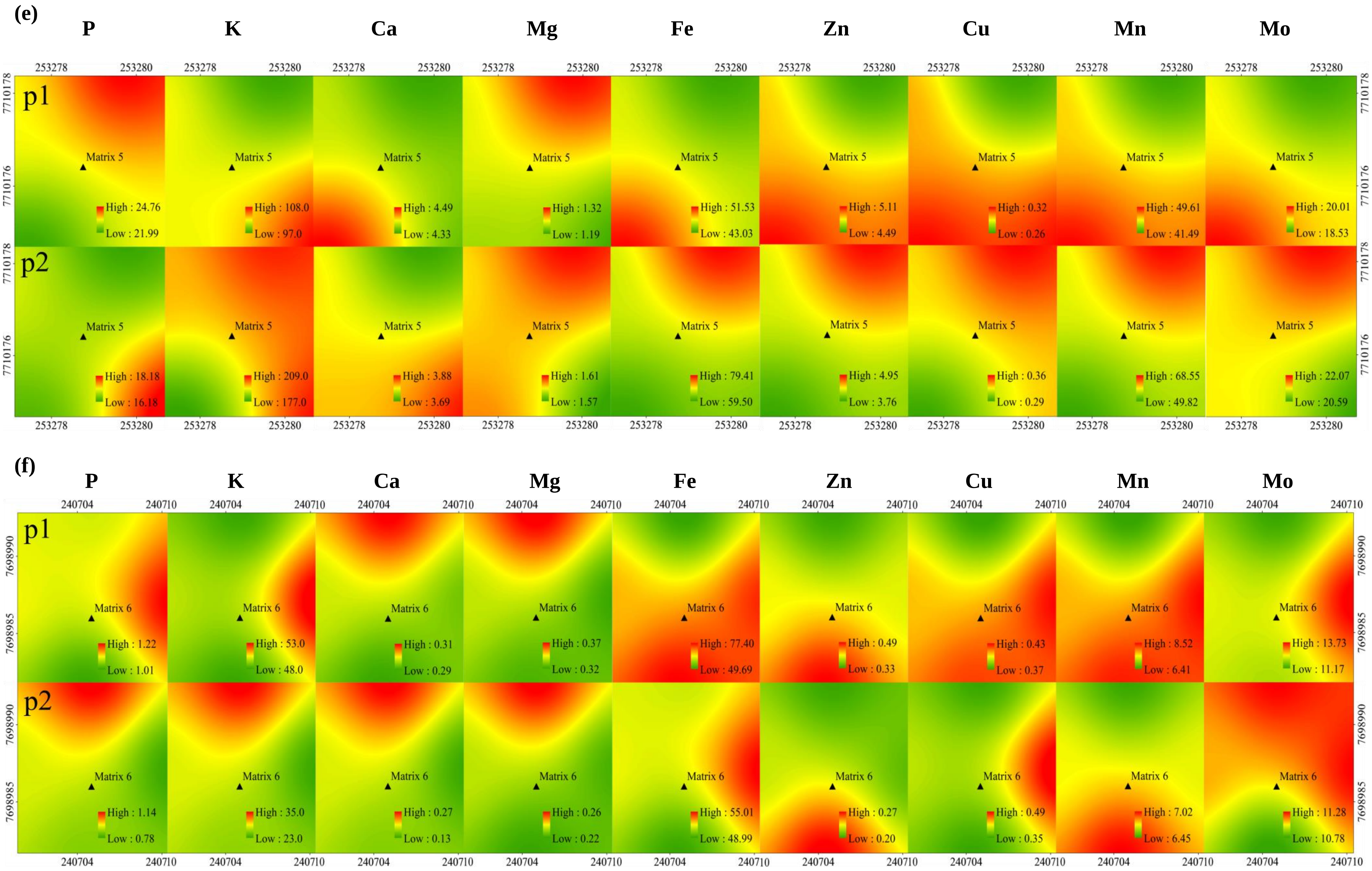
(e)
P
K
Ca
Mg
Fe
Zn
Cu
Mn
Mo
253278
253280
7710178
7710176
p1
Matrix 5
High : 24.76
Low : 21.99
High : 108.0
Low : 97.0
High : 4.49
Low : 4.33
High : 1.32
Low : 1.19
High : 51.53
Low : 43.03
High : 5.11
Low : 4.49
High : 0.32
Low : 0.26
High : 49.61
Low : 41.49
High : 20.01
Low : 18.53
p2
High : 18.18
Low : 16.18
High : 209.0
Low : 177.0
High : 3.88
Low : 3.69
High : 1.61
Low : 1.57
High : 79.41
Low : 59.50
High : 4.95
Low : 3.76
High : 0.36
Low : 0.29
High : 68.55
Low : 49.82
High : 22.07
Low : 20.59
(f)
P
K
Ca
Mg
Fe
Zn
Cu
Mn
Mo
240704
240710
7698990
7698985
p1
Matrix 6
High : 1.22
Low : 1.01
High : 53.0
Low : 48.0
High : 0.31
Low : 0.29
High : 0.37
Low : 0.32
High : 77.40
Low : 49.69
High : 0.49
Low : 0.33
High : 0.43
Low : 0.37
High : 8.52
Low : 6.41
High : 13.73
Low : 11.17
p2
High : 1.14
Low : 0.78
High : 35.0
Low : 23.0
High : 0.27
Low : 0.13
High : 0.26
Low : 0.22
High : 55.01
Low : 48.99
High : 0.27
Low : 0.20
High : 0.49
Low : 0.35
High : 7.02
Low : 6.45
High : 11.28
Low : 10.78

Supplementary Figure. S1: Soil maps at depths of 0–20 cm (p1) and 20–40 cm (p2) representing the composition of the soil where the mother trees of *L. pisonis* are located: (a) tree 1, (b) tree 2, (c) tree 3, (d) tree 4, (e) tree 5 and (f) tree 6, emphasizing nutrients P (mg dm^{-3}), K (mg dm^{-3}), Ca ($cmol_c$ dm^{-3}), Mg ($cmol_c$ dm^{-3}), Fe (mg dm^{-3}), Zn (mg dm^{-3}), Mn (mg dm^{-3}), Cu (mg dm^{-3}), and organic matter - Mo (g kg^{-1}).

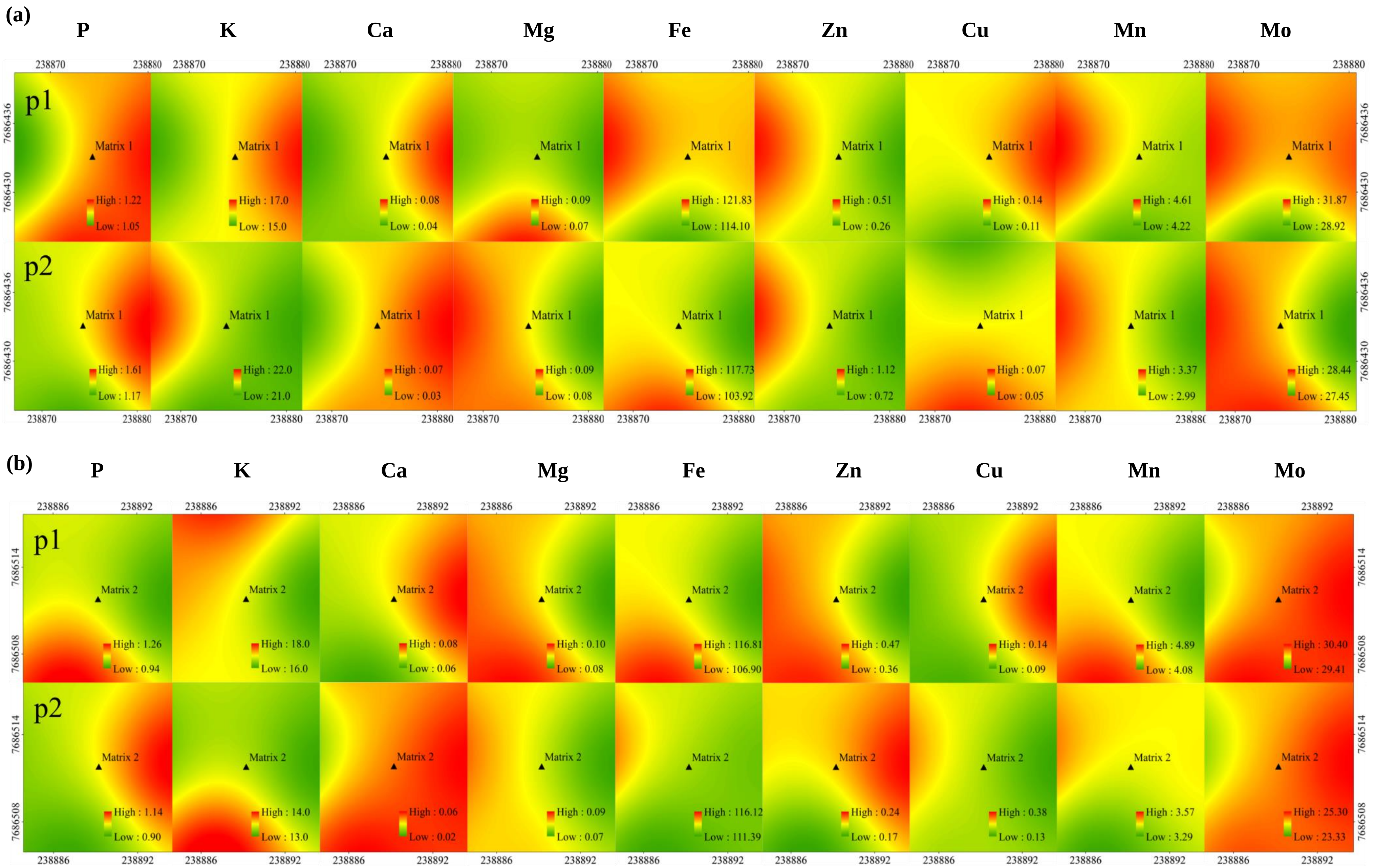

(a)
P
K
Ca
Mg
Fe
Zn
Cu
Mn
Mo
238870
238880
7686436
7686430
p1
p2
Matrix 1
High : 1.22
Low : 1.05
High : 17.0
Low : 15.0
High : 0.08
Low : 0.04
High : 0.09
Low : 0.07
High : 121.83
Low : 114.10
High : 0.51
Low : 0.26
High : 0.14
Low : 0.11
High : 4.61
Low : 4.22
High : 31.87
Low : 28.92
High : 1.61
Low : 1.17
High : 22.0
Low : 21.0
High : 0.07
Low : 0.03
High : 0.09
Low : 0.08
High : 117.73
Low : 103.92
High : 1.12
Low : 0.72
High : 0.07
Low : 0.05
High : 3.37
Low : 2.99
High : 28.44
Low : 27.45
(b)
P
K
Ca
Mg
Fe
Zn
Cu
Mn
Mo
238886
238892
7686514
7686508
p1
p2
Matrix 2
High : 1.26
Low : 0.94
High : 18.0
Low : 16.0
High : 0.08
Low : 0.06
High : 0.10
Low : 0.08
High : 116.81
Low : 106.90
High : 0.47
Low : 0.36
High : 0.14
Low : 0.09
High : 4.89
Low : 4.08
High : 30.40
Low : 29.41
High : 1.14
Low : 0.90
High : 14.0
Low : 13.0
High : 0.06
Low : 0.02
High : 0.09
Low : 0.07
High : 116.12
Low : 111.39
High : 0.24
Low : 0.17
High : 0.38
Low : 0.13
High : 3.57
Low : 3.29
High : 25.30
Low : 23.33

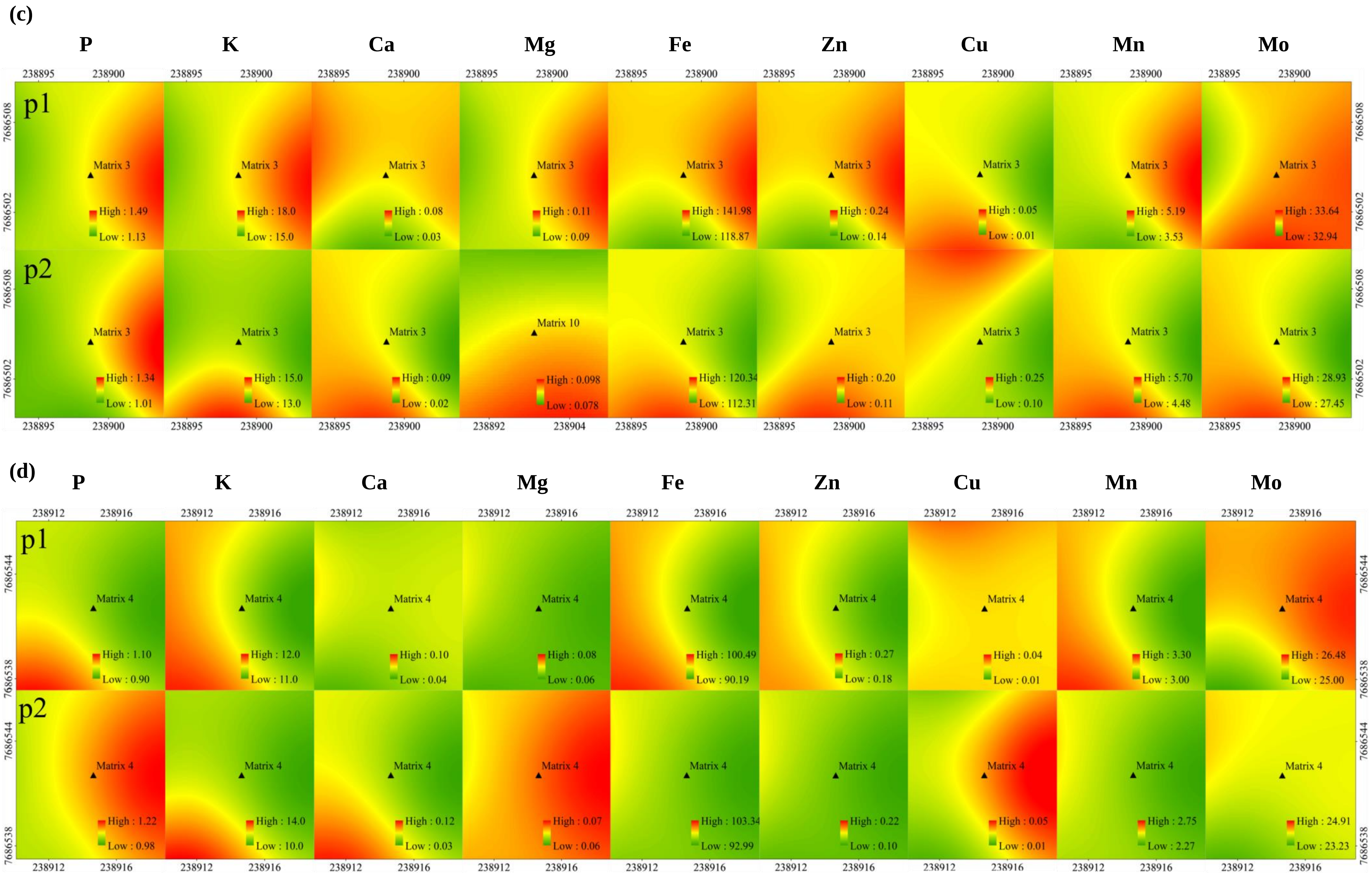
(c)
P
K
Ca
Mg
Fe
Zn
Cu
Mn
Mo
p1
p2
238895
238900
238892
238904
7686508
7686502
Matrix 3
Matrix 10
High : 1.49
Low : 1.13
High : 18.0
Low : 15.0
High : 0.08
Low : 0.03
High : 0.11
Low : 0.09
High : 141.98
Low : 118.87
High : 0.24
Low : 0.14
High : 0.05
Low : 0.01
High : 5.19
Low : 3.53
High : 33.64
Low : 32.94
High : 1.34
Low : 1.01
High : 15.0
Low : 13.0
High : 0.09
Low : 0.02
High : 0.098
Low : 0.078
High : 120.34
Low : 112.31
High : 0.20
Low : 0.11
High : 0.25
Low : 0.10
High : 5.70
Low : 4.48
High : 28.93
Low : 27.45
(d)
P
K
Ca
Mg
Fe
Zn
Cu
Mn
Mo
p1
p2
238912
238916
7686544
7686538
Matrix 4
High : 1.10
Low : 0.90
High : 12.0
Low : 11.0
High : 0.10
Low : 0.04
High : 0.08
Low : 0.06
High : 100.49
Low : 90.19
High : 0.27
Low : 0.18
High : 0.04
Low : 0.01
High : 3.30
Low : 3.00
High : 26.48
Low : 25.00
High : 1.22
Low : 0.98
High : 14.0
Low : 10.0
High : 0.12
Low : 0.03
High : 0.07
Low : 0.06
High : 103.34
Low : 92.99
High : 0.22
Low : 0.10
High : 0.05
Low : 0.01
High : 2.75
Low : 2.27
High : 24.91
Low : 23.23

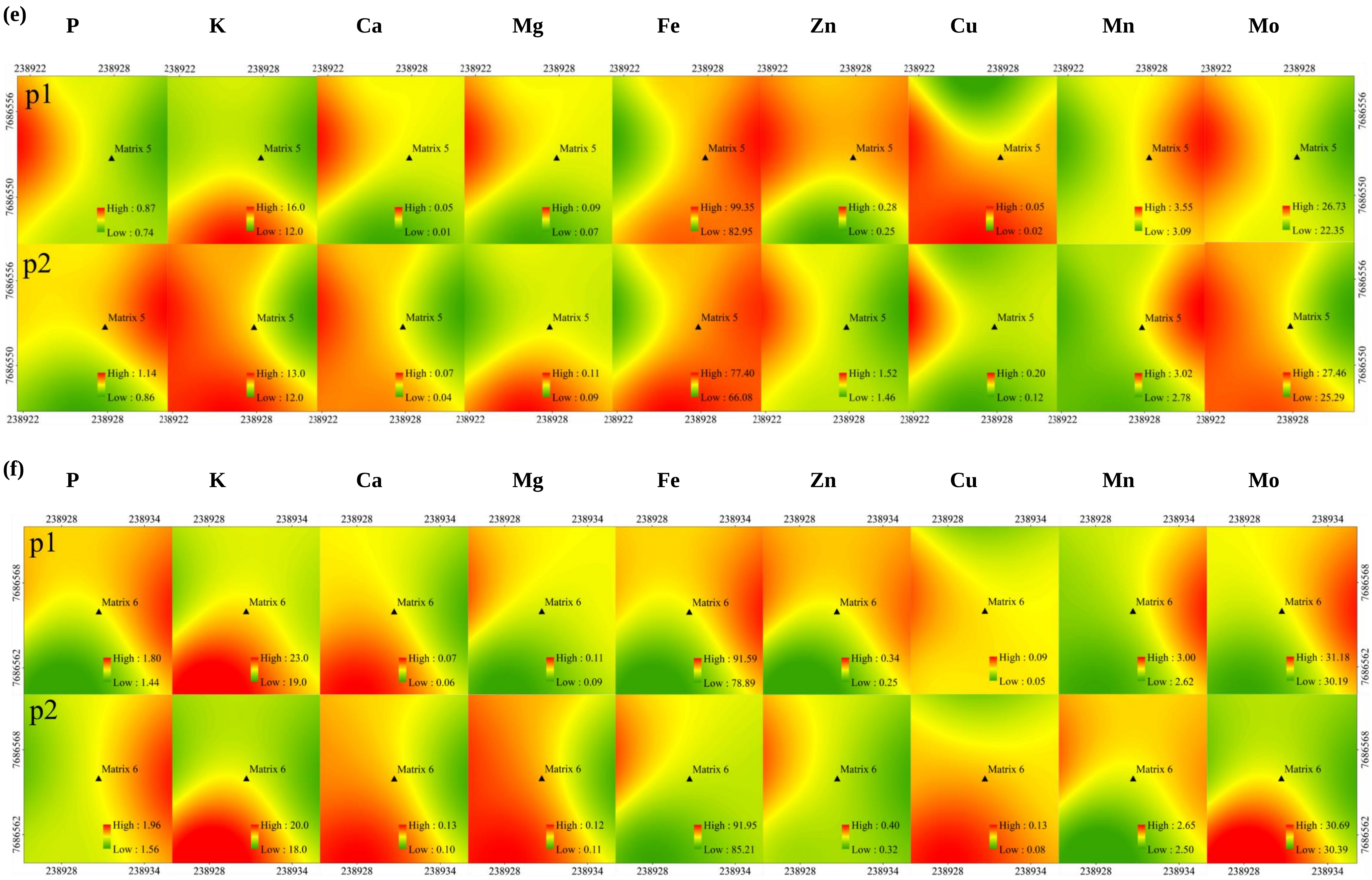
(e)
P
K
Ca
Mg
Fe
Zn
Cu
Mn
Mo
238922
238928
7686556
7686550
p1
p2
Matrix 5
High : 0.87
Low : 0.74
High : 16.0
Low : 12.0
High : 0.05
Low : 0.01
High : 0.09
Low : 0.07
High : 99.35
Low : 82.95
High : 0.28
Low : 0.25
High : 0.05
Low : 0.02
High : 3.55
Low : 3.09
High : 26.73
Low : 22.35
High : 1.14
Low : 0.86
High : 13.0
Low : 12.0
High : 0.07
Low : 0.04
High : 0.11
Low : 0.09
High : 77.40
Low : 66.08
High : 1.52
Low : 1.46
High : 0.20
Low : 0.12
High : 3.02
Low : 2.78
High : 27.46
Low : 25.29
(f)
P
K
Ca
Mg
Fe
Zn
Cu
Mn
Mo
238928
238934
7686568
7686562
p1
p2
Matrix 6
High : 1.80
Low : 1.44
High : 23.0
Low : 19.0
High : 0.07
Low : 0.06
High : 0.11
Low : 0.09
High : 91.59
Low : 78.89
High : 0.34
Low : 0.25
High : 0.09
Low : 0.05
High : 3.00
Low : 2.62
High : 31.18
Low : 30.19
High : 1.96
Low : 1.56
High : 20.0
Low : 18.0
High : 0.13
Low : 0.10
High : 0.12
Low : 0.11
High : 91.95
Low : 85.21
High : 0.40
Low : 0.32
High : 0.13
Low : 0.08
High : 2.65
Low : 2.50
High : 30.69
Low : 30.39

Supplementary Figure. S2: Soil maps at depths of 0–20 cm (p1) and 20–40 cm (p2) representing the composition of the soil where the mother trees of *L. lanceolata* are located: (a) tree 1, (b) tree 2, (c) tree 3, (d) tree 4, (e) tree 5 and (f) tree 6, emphasizing nutrients P (mg dm^{-3}), K (mg dm^{-3}), Ca ($cmol_c$ dm^{-3}), Mg ($cmol_c$ dm^{-3}), Fe (mg dm^{-3}), Zn (mg dm^{-3}), Mn (mg dm^{-3}), Cu (mg dm^{-3}), and organic matter - Mo (g kg^{-1}).

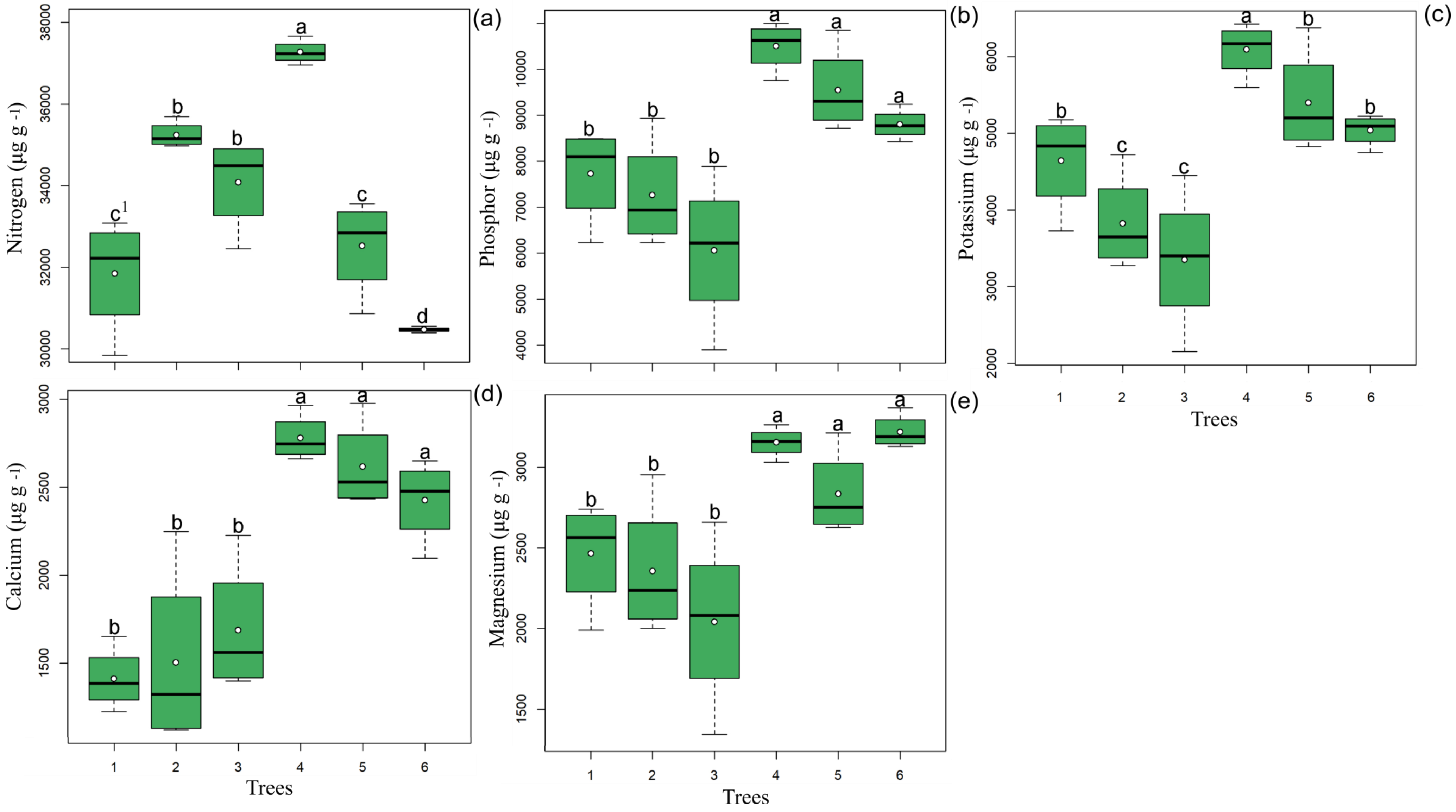
(a)
Nitrogen (µg g -1)
38000
36000
34000
32000
30000
a
b
b
c1
c
d
(b)
Phosphor (µg g -1)
10000
9000
8000
7000
6000
5000
4000
(c)
Potassium (µg g -1)
6000
5000
4000
3000
2000
1 2 3 4 5 6
Trees
(d)
Calcium (µg g -1)
3000
2500
2000
1500
1 2 3 4 5 6
Trees
(e)
Magnesium (µg g -1)
3000
2500
2000
1500
1 2 3 4 5 6
Trees

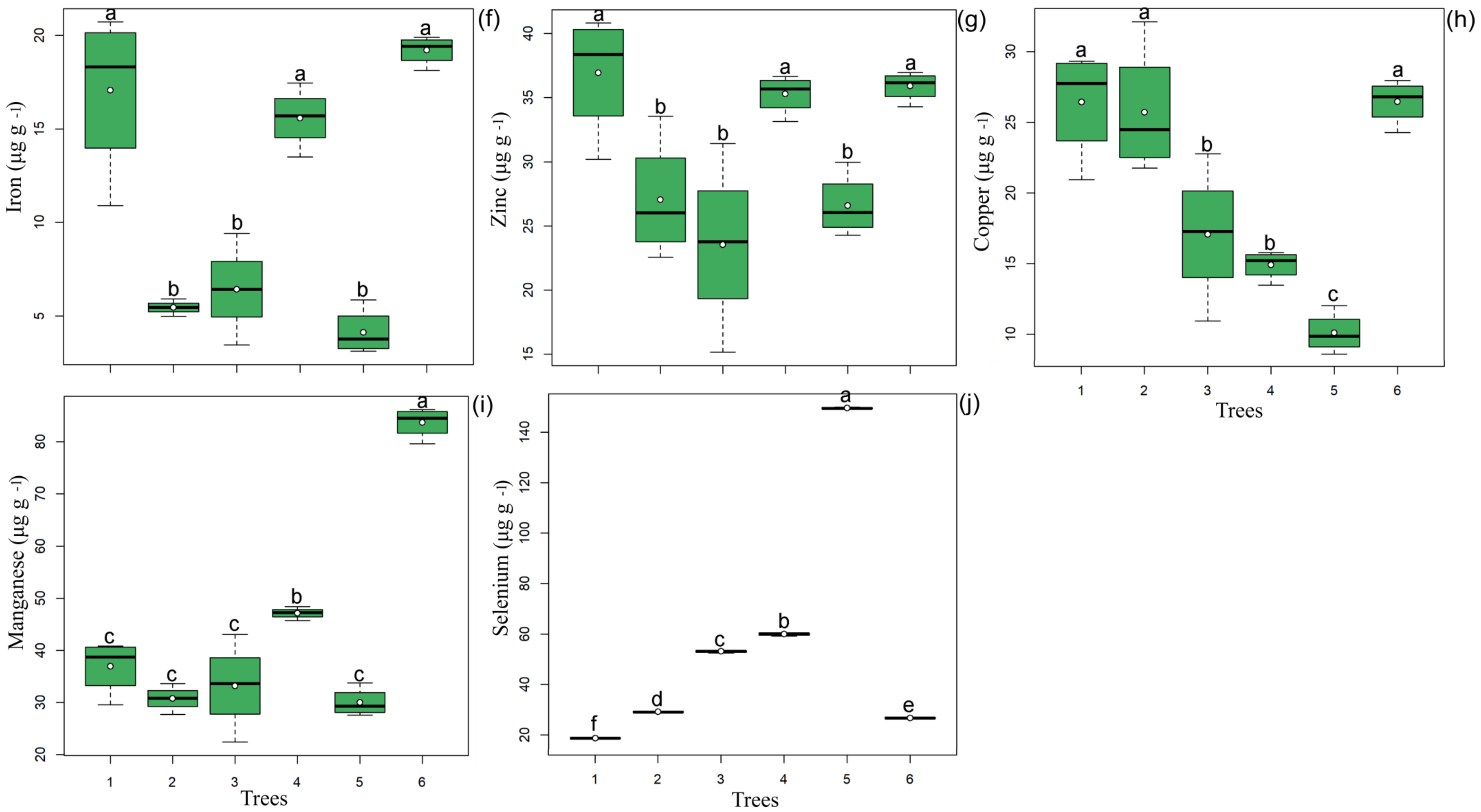

(f)
Iron (µg g -1)
(g)
Zinc (µg g -1)
(h)
Copper (µg g -1)
(i)
Manganese (µg g -1)
(j)
Selenium (µg g -1)
Trees

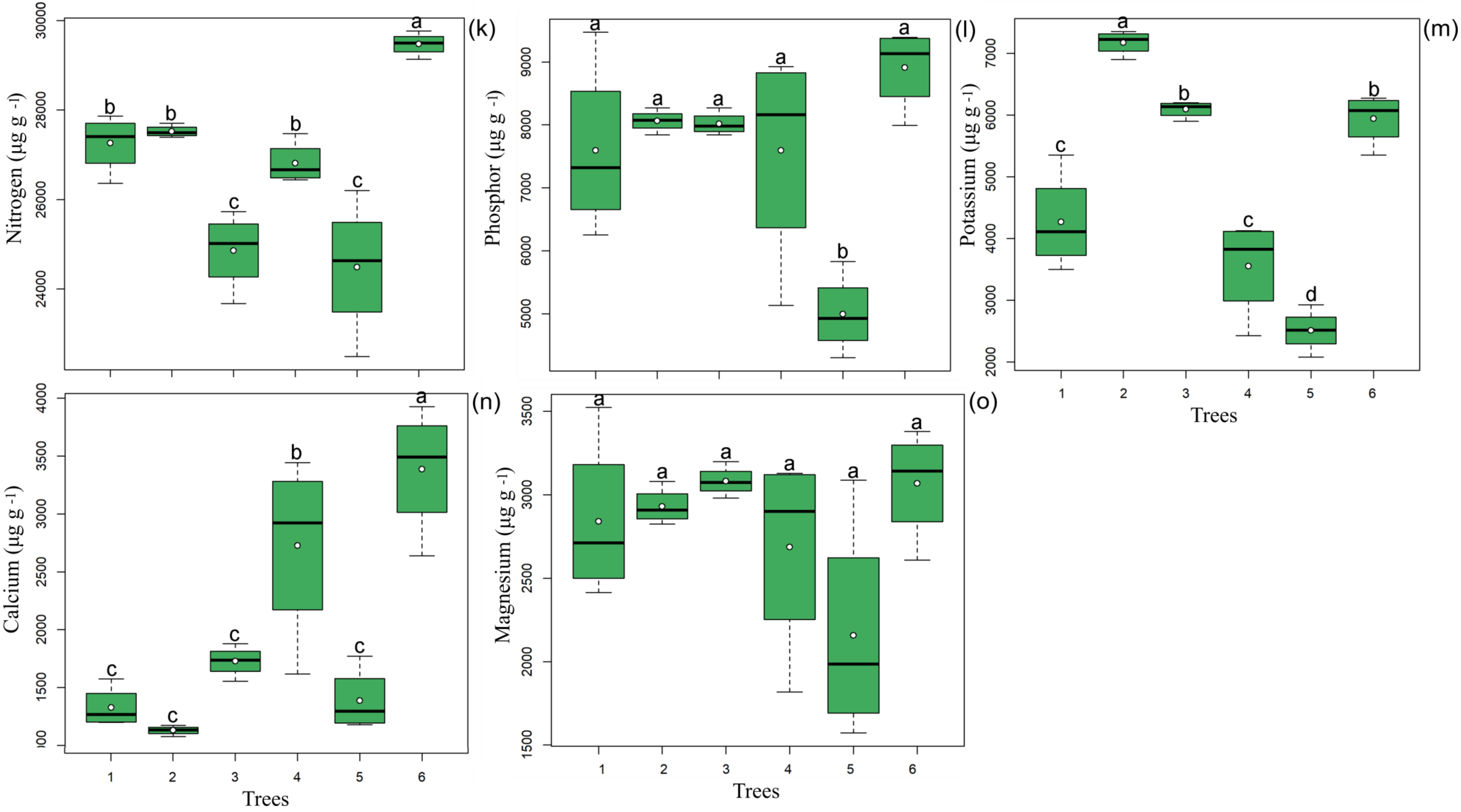
(k)
Nitrogen (µg g -1)
(l)
Phosphor (µg g -1)
(m)
Potassium (µg g -1)
Trees
(n)
Calcium (µg g -1)
Trees
(o)
Magnesium (µg g -1)
Trees

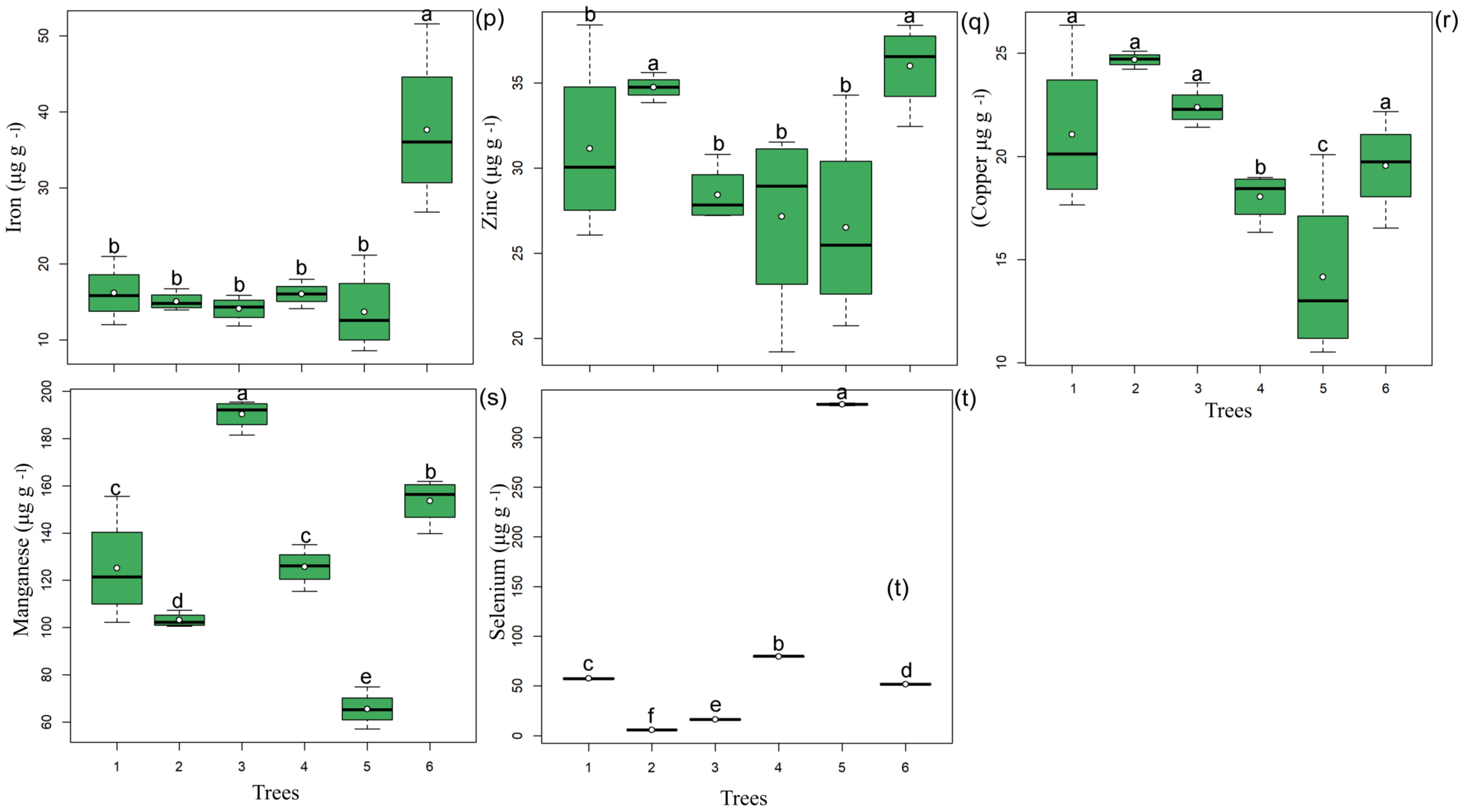

Supplementary Figure. S3: Box plots summarizing the content of N, P, K, Ca, Mg, Fe, Zn, Cu, Mn, and Se in the nuts of *L. pisonis* (a–j) and *L. lanceolata* (k–t).[1] Means followed by the same letter belong to the same group of averages based on the Scott-Knott group of averages test ($p \leq 0.05$).

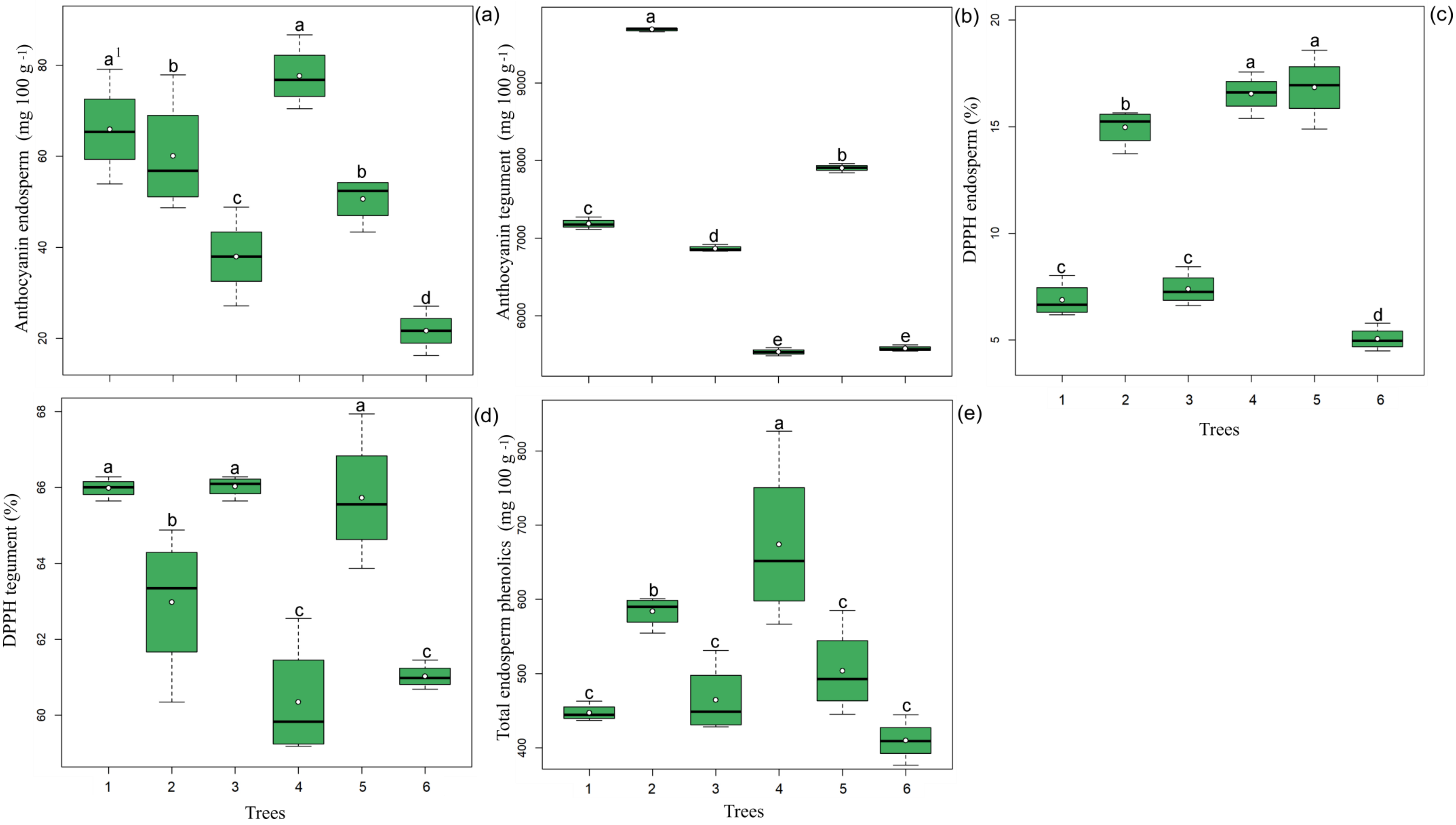

(a)
(b)
(c)
(d)
(e)
Anthocyanin endosperm (mg 100 g -1)
Anthocyanin tegument (mg 100 g -1)
DPPH endosperm (%)
DPPH tegument (%)
Total endosperm phenolics (mg 100 g -1)
Trees

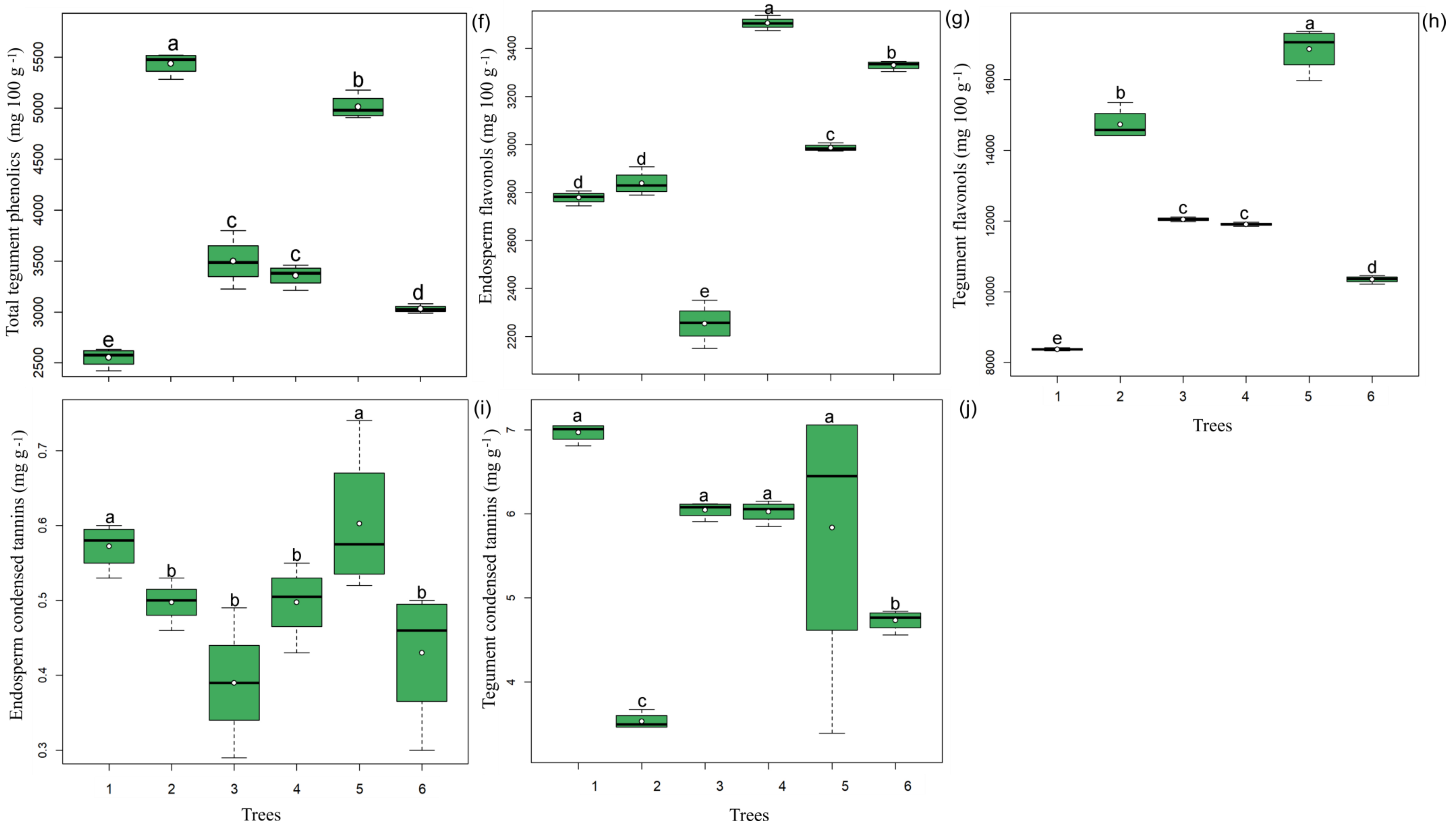

(f)
(g)
(h)
(i)
(j)
Total tegument phenolics (mg 100 g -1)
Endosperm flavonols (mg 100 g-1)
Tegument flavonols (mg 100 g-1)
Endosperm condensed tannins (mg g-1)
Tegument condensed tannins (mg g-1)
Trees
1 2 3 4 5 6
e a c c b d
d d e a c b
e b c c a d
a b b b a b
a c a a a b

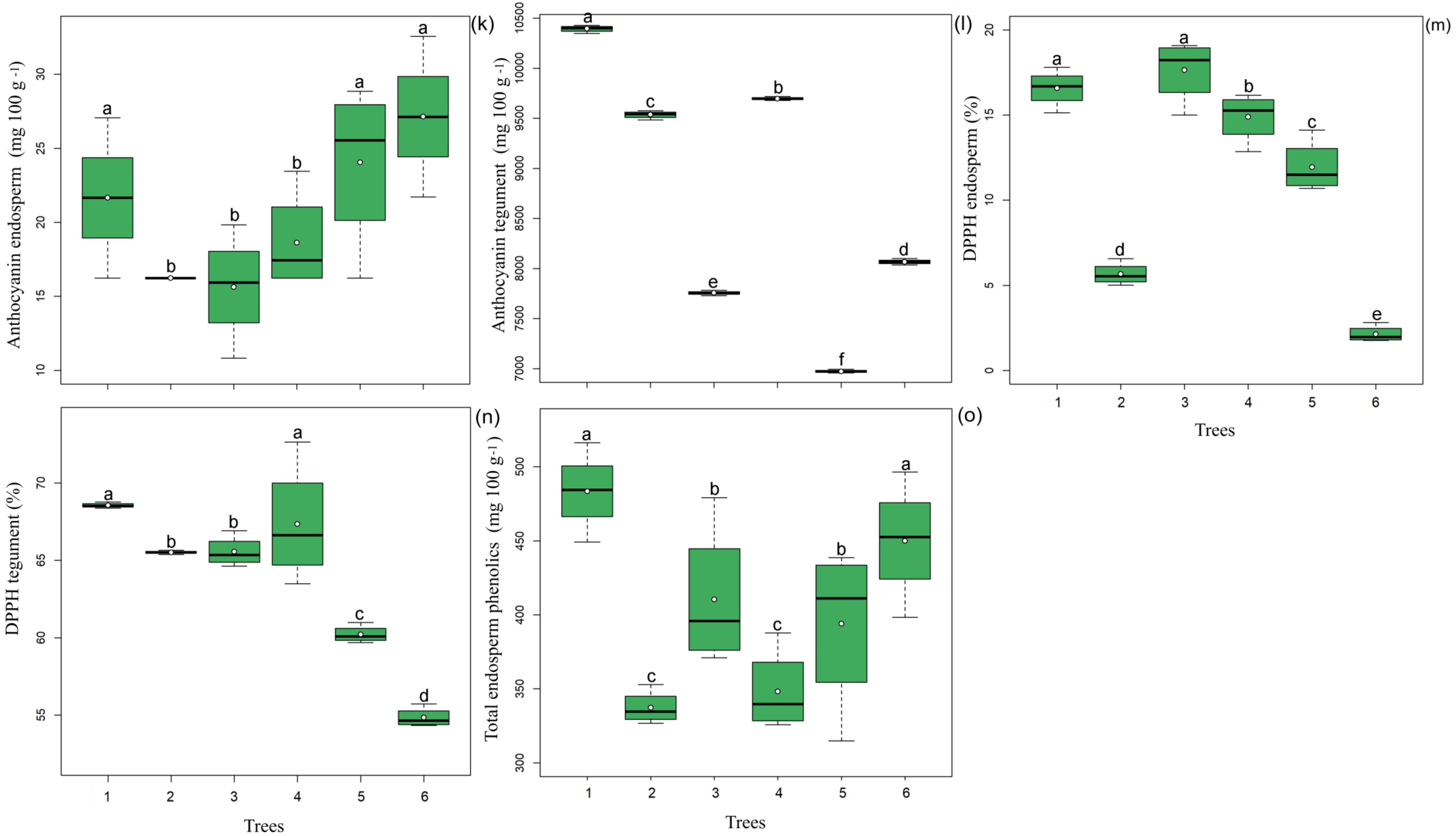
(k)
Anthocyanin endosperm (mg 100 g -1)
10
15
20
25
30
(l)
Anthocyanin tegument (mg 100 g -1)
7000
7500
8000
8500
9000
9500
10000
10500
(m)
DPPH endosperm (%)
0
5
10
15
20
(n)
DPPH tegument (%)
55
60
65
70
(o)
Total endosperm phenolics (mg 100 g-1)
300
350
400
450
500
1
2
3
4
5
6
Trees

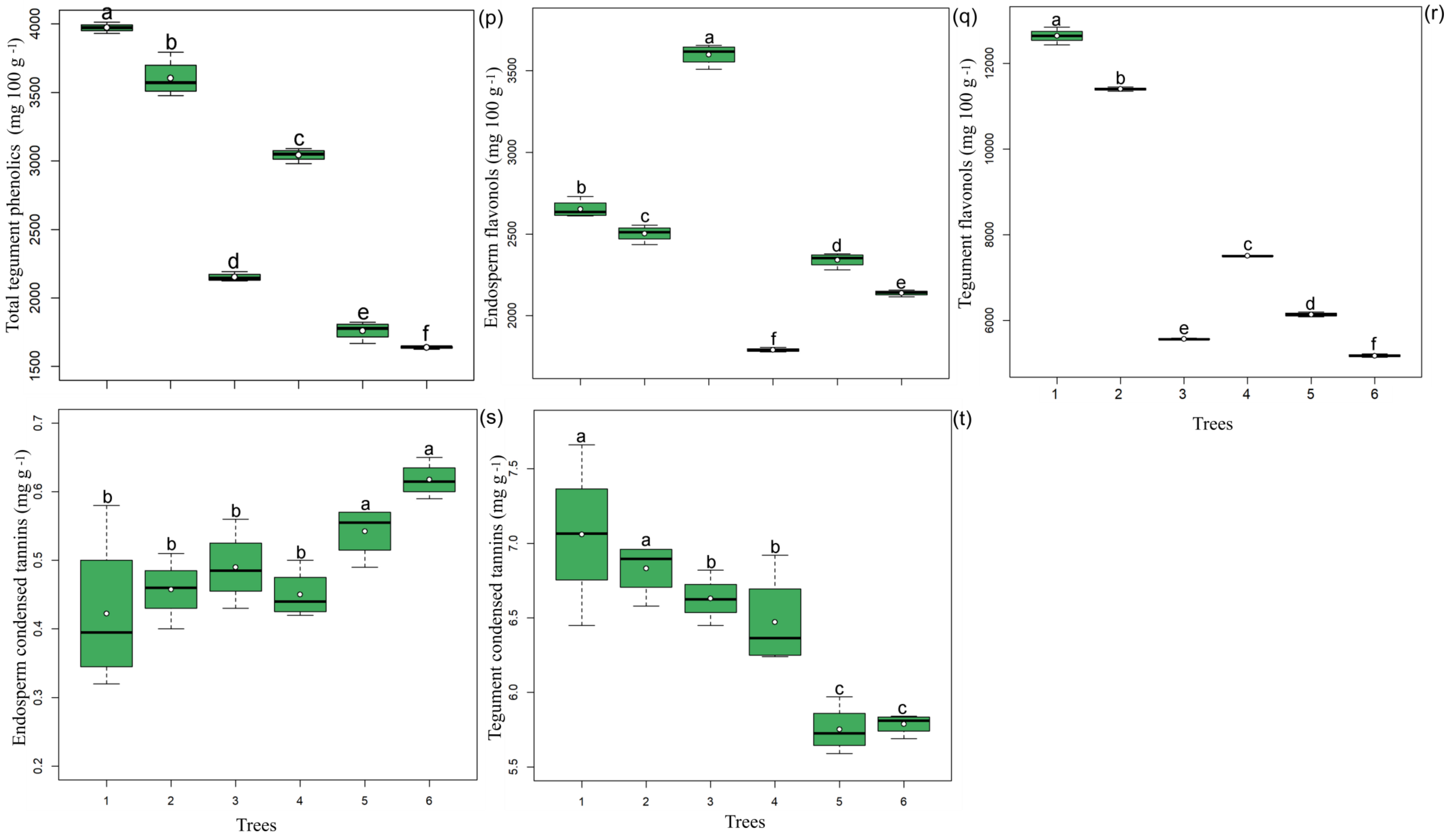

Supplementary Figure. S4: Box plots summarizing the content of antioxidant compounds in the nuts of *L. pisonis* (a–j) and *L. lanceolata* (k–t). [1]Means followed by the same letter belong to the same group of averages based on the Scott-Knott group of averages test ($p \leq 0.05$).
