# Supplementary material for: Functional Fruit Trees from the Atlantic and Amazon Forests: Selection of Potential Chestnut Trees Rich in Antioxidants, Nutrients, and Fatty Acids
Source: Foods. 2023 Dec 9;12(24):4422. doi: 10.3390/foods12244422 (PMC10743210; doi:10.3390/foods12244422)
Supplement: Supplementary file 1 [file foods-12-04422-s001.zip › Table suplementar.pdf]

Table suplementary S1: Nutrients present in the leaves of *L. pisonis* and *L. lanceolata* trees. [1]Means followed by the same letter in the column belong to the same group of averages based on the Scott-Knott group of averages test ($p \leq 0.05$). ns = not significant.

Species	Trees	Leaf nutrients (μg g^{-1})									
		N	P	K	Ca	Mg	S	Zn	Fe	Mn	Cu
L. lanceolata	1	33.58ns	1.77c	4.73b	3.50c	1.79a	2.66a	17.76c	63.10b	99.42c	15.96c
	2	27.15ns	2.23b	2.99c	4.91b	1.96a	1.71b	22.74b	79.66a	139.33a	18.52b
	3	29.82ns	1.76c	3.30c	2.55d	1.88a	2.43a	18.64c	82.11a	134.96a	18.61b
	4	30.13ns	1.82c	4.53b	4.67b	1.57b	1.93b	20.31b	56.49b	124.75b	14.94d
	5	34.85ns	2.80a	6.43a	2.31d	1.33c	2.74a	27.44a	77.64a	33.50d	21.14a
	6	31.63ns	2.38b	6.00a	5.39a	1.75a	2.34a	21.63b	74.66a	109.17c	16.93c
L. pisonis	1	23.12b	1.58a	3.23e	33.75a	2.91a	1.54c	8.20b	82.71b	377.28a	6.88d
	2	26.34a	1.25b	3.89d	27.53b	2.45b	2.16b	6.43b	77.66b	250.29c	7.16d
	3	18.64c	1.30b	8.20a	4.90e	1.09d	1.29c	9.79a	44.37b	40.43f	9.77c
	4	23.68b	1.62a	6.24b	17.58c	2.54b	2.34b	9.65a	102.31b	202.89d	7.57d
	5	21.80b	1.83a	7.99a	12.32d	1.80c	2.33b	11.77a	150.46a	73.45e	10.64b
	6	22.99b	1.32b	5.70c	11.43d	1.81c	3.33a	9.48a	73.80b	332.62b	13.48a
